# Targeting Ferroptosis in Tumors: Novel Marine-Derived Compounds as Regulators of Lipid Peroxidation and GPX4 Signaling

**DOI:** 10.3390/md23060258

**Published:** 2025-06-19

**Authors:** Yimao Wu, Xiaoyan Chen, Zichang Chen, Yunqi Ma

**Affiliations:** 1 School of Pharmacy, Binzhou Medical University, Yantai 264003, China; wuyimao_doctor@gdmu.edu.cn; 2 Second Clinical Medical College, Guangdong Medical University, Dongguan 523808, China; xiaoyanchen_doctor@163.com (X.C.); zichangchen0@gmail.com (Z.C.)

**Keywords:** ferroptosis, marine-derived compounds, lipid peroxidation, GPX4, ACSL4(1), lipid metabolism, targeted therapy

## Abstract

This article reviews the mechanisms by which marine natural products regulate ferroptosis and their potential applications in tumor therapy. Ferroptosis is a form of programmed cell death driven by iron-dependent lipid peroxidation, characterized primarily by the accumulation of lipid peroxides and the failure of antioxidant defense systems. Due to their unique chemical structural diversity, marine natural products demonstrate significant advantages in regulating the ferroptosis pathway. Studies showed that marine compounds target key molecules such as glutathione peroxidase 4 (GPX4) and long-chain acyl-CoA synthetase 4 (ACSL4(a)) ACSL4(1) to modulate lipid peroxidation and iron metabolism, inducing ferroptosis in tumor cells and reshaping the tumor microenvironment (TME). In addition, marine compounds can enhance anti-tumor effects by activating immune responses. Although marine compounds hold great potential in regulating ferroptosis, their clinical translation faces challenges such as low bioavailability and tumor type dependency. Future research needs to integrate multi-omics techniques to further analyze the mechanisms of marine compounds and develop precision therapeutic strategies based on marine compounds to overcome the bottlenecks in ferroptosis therapy.

## 1. Introduction

**Figure 1:**
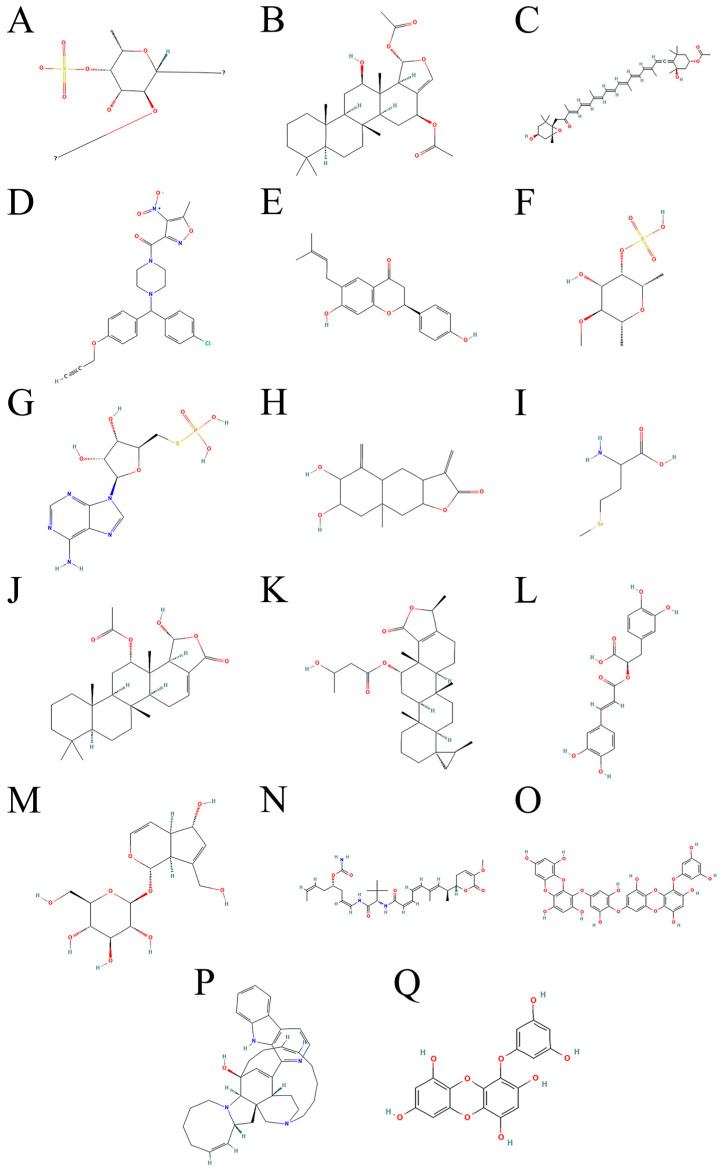
The chemical structures of all compounds mentioned in the manuscript. (**A**) ACSL4(1). (**B**) Heteronemin(2). (**C**) Fucoxanthin(3). (**D**) ML210(4). (**E**) Bavachin(5). (**F**) Fucoidan sulfate(6). (**G**) ASMP(7). (**H**) Sesquiterpene lactone(8). (**I**) Selenomethionine(9). (**J**) Scalarin(10). (**K**) Honulactone A(11). (**L**) Rosmarinic acid(12). (**M**) Aucubin(13). (**N**) Plocabulin(14). (**O**) Dieckol(15). (**P**) Manzamine A(16). (**Q**) Eckol(17).

Ferroptosis is a new type of cell death, which is different from apoptosis, necrosis, pyroptosis, and autophagy [1]. Ferroptosis is a newly defined form of regulatory cell death (RCD) characterized by the accumulation of iron-overload reactive oxygen species (ROS) and lipid peroxidation [2,3,4]. As a form of programmed cell death driven by iron-dependent lipid peroxidation, ferroptosis’s core regulatory network provides a new target for cancer treatment. Marine natural products have shown significant advantages in regulating ferroptosis pathways due to their unique chemical structural diversity: terpenoids can degrade glutathione peroxidase 4 (GPX4) or inhibit long-chain acyl-CoA synthetase 4 (ACSL4(1)) (see Figure 1A for chemical structure) activity, polyphenols can dually regulate lipid peroxidation and antioxidant defense, and sulfur compounds can covalently modify a GPX4 active site, which constitute a multi-dimensional intervention strategy [5]. Recent studies revealed that marine compounds not only directly induce ferroptosis of tumor cells but also can reprogram the lipid metabolism phenotype of tumor-associated macrophages (TAMs) to form a ferroptosis-promoting immune microenvironment. Integrating lipidomics and single-cell sequencing technology, it was found that marine compounds significantly enhanced the lipid peroxidation cascade by reshaping the phospholipid composition of the tumor cell membrane and regulating mitochondrial REDOX homeostasis. Preclinical models confirmed that marine-derived nano-delivery systems can break through bioavailability limitations, and Clustered Regularly Interspaced Short Palindromic Repeats-Cas9 (CRISPR-Cas9)-based target verification technology provides a precise tool for compound optimization. This review systematically analyzed the molecular mechanism of marine natural products regulating ferroptosis through lipid peroxidation and GPX4 signaling pathways, emphasizing the structure–activity relationship between their structural properties and functional targets, and proposed a multi-omics-based precise intervention strategy, which provides an innovative perspective for the development of the next generation of ferroptosis-targeted anticancer drugs.

### 1.1. Biology of Ferroptosis

**Figure 2:**
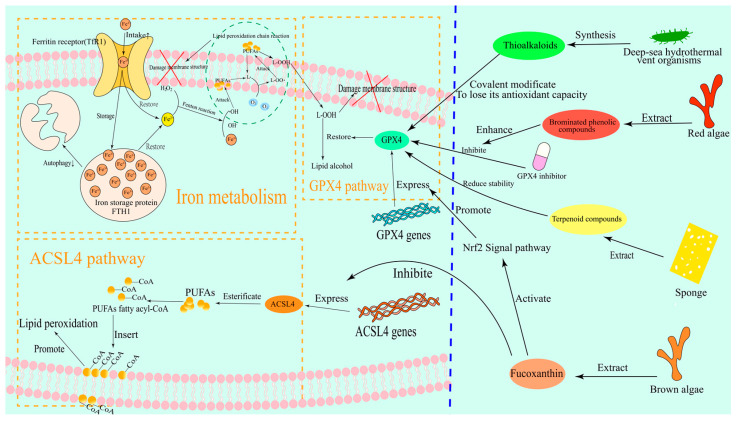
Iron metabolism and the interaction of antioxidant defense mechanisms and their regulatory network. Figure 2 illustrates the interaction mechanisms of iron metabolism, antioxidant defense, and related signaling pathways. It involves the uptake, storage, and metabolism of iron ions as well as their relationship with the antioxidant enzyme GPX4, the nuclear factor erythroid 2-related factor 2 (Nrf2) signaling pathway, and the ACSL4(1) signaling pathway. Additionally, it demonstrates the potential effects of bioactive compounds derived from deep-sea hydrothermal vent organisms, red algae, brown algae, and other sources of iron metabolism and antioxidant defense.

Ferroptosis is a unique form of iron-dependent cell death that is characterized by the abnormal accumulation of lipid peroxides and failure of the antioxidant defense system. This process shows a complex regulatory network and therapeutic potential in tumor biology [6]. Lipid peroxides (PLOOH) are considered to be the key mediators of ferroptosis due to their ability to cause damage to the lipid bilayer of the cell membrane [7]. In addition, the degradation products of lipid peroxides can also cause additional toxicity by altering the structure and function of nucleic acids [8]. Firstly, the initiation of ferroptosis depends on the accumulation of free iron in the cell. Iron ions catalyze the lipid peroxidation chain reaction through the Fenton reaction, generate a large number of reactive oxygen species (ROS), and cause membrane structural damage [9,10,11]. Notably, the imbalance of iron metabolism not only involves the enhanced extracellular iron uptake mediated by transferrin receptor (TfR1) but is also closely related to the autophagic degradation of iron storage proteins such as ferritin heavy chain 1 (FTH1). This dual regulation makes tumor cells abnormally sensitive to iron overload [12]. The mechanism of how iron metabolism pathways cause membrane structural damage is shown in Figure 2.

At the level of lipid metabolism, ACSL4(1) is considered to be the key executor of ferroptosis [5,13]. ACSL4(1) significantly increases the sensitivity of cell membranes to peroxidation by esterifying polyunsaturated fatty acids (PUFAs) to acyl-CoA derivatives and promoting the synthesis of PUFAs-containing phospholipids, such as phosphatidylethanolamine [14,15]. The mechanism of the ACSL4(1) pathway related to membrane structural damage is shown in Figure 2. For example, it was found that the ubiquitination modification of ACSL4(1) (such as UBR5-mediated ubiquitination at K388, K498, and K690 sites) directly leads to its protein degradation, thereby blocking the biosynthesis of PUFA phospholipids and ultimately inhibiting ferroptosis [12]. This finding revealed the pivotal role of ACSL4(1) in the regulation of ferroptosis and also provided a theoretical basis for cancer treatment strategies targeting ACSL4(1).

In contrast to the pro-ferroptosis role of ACSL4(1), GPX4 is the core intracellular antioxidant enzyme against lipid peroxidation [16]. GPX4 utilizes reduced glutathione (GSH) to reduce lipid peroxides (L-OOH) to non-toxic lipid alcohols (L-OH), thereby maintaining the REDOX homeostasis of membrane lipids [8,17,18]. The mechanism of the GPX4 pathway in causing membrane structural damage is shown in Figure 2. GSH is an important member of the cellular antioxidant system, and its synthesis depends on cysteine originating from the system Xc^−^/GSH/GPX4 axis [19]. Therefore, the functional inhibition of system Xc^−^ (cysteinyl/glutamate antiporter) or down-regulation of the rate-limiting enzymes of GSH synthesis such as GCLC in tumor cells leads to the loss of GPX4 activity, which in turn induces irreversible lipid peroxidation damage [20,21]. For example, in colorectal cancer cells, oxaliplatin significantly enhanced the sensitivity of cells to ferroptosis by inhibiting GPX4 activity, which could be reversed by ferroptosis inhibitors such as Ferrostatin-1 [12,22]. This phenomenon suggests that the functional status of GPX4 is a key molecular switch that determines the susceptibility of tumor cells to ferroptosis.

The relationship between ferroptosis and tumor drug resistance has become a research hotspot in recent years. Traditional chemotherapy drugs (such as cisplatin) or targeted therapy often fail due to the acquired apoptotic resistance of tumor cells, and ferroptosis provides an alternative killing mechanism that bypasses the apoptotic pathway [23,24]. For example, in triple-negative breast cancer, epithelial–mesenchymal transition (EMT)-related drug resistance phenotypes are closely associated with the up-regulation of ACSL4(1) expression and inhibition of GPX4 activity, and induction of ferroptosis can significantly inhibit the growth of such resistant tumors [25,26]. In addition, immune escape in the tumor microenvironment (TME) may also be related to the regulation of ferroptosis. Studies found that TAMs can regulate local iron homeostasis by secreting transferrin or hepcidin, thereby affecting the ferroptosis threshold of tumor cells [27,28]. This interaction between iron metabolism and immune microenvironment provides a new theoretical framework for combining ferroptosis inducers with immune checkpoint inhibitors.

On the basis of existing studies, we proposed that, although ACSL4(1) and GPX4 were established as the core regulatory molecules of ferroptosis, their functional heterogeneity in different tumor types still needs to be further elucidated. For example, some tumors (such as liver cancer) may resist ferroptosis induced by GPX4 inactivation by activating alternative lipid repair pathways (such as the ferroptosis suppressor protein 1 (FSP1)/Coenzyme Q (CoQ)10 system). However, whether the ACSL4(1) expression level can be used as a biomarker to predict the efficacy of ferroptosis inducers still needs to be verified in large-scale clinical trials [13,29]. In addition, the relationship between ferroptosis and cancer stem cells’ (CSCs) properties is not clearly established—whether CSCs maintain their antioxidant capacity through the up-regulation of the iron chelation pathway or lipid desaturation enzymes such as Stearoyl-CoA desaturase 1 (SCD1) may be an important direction for future research. In view of these scientific problems, the development of multi-dimensional intervention strategies that can simultaneously target iron metabolism, lipid peroxidation, and antioxidant pathways will be the key to breaking through the bottleneck of current ferroptosis therapy.

### 1.2. Marine Natural Products in Drug Discovery

Marine natural products exhibit unique chemical diversity and biological activities in drug discovery, especially in regulating ferroptosis-related signaling pathways. The core mechanism of ferroptosis involves the accumulation of lipid peroxidation and the dysregulation of the GPX4 antioxidant system. Marine organisms have evolved special metabolic pathways to adapt to extreme environments for a long time, making secondary metabolites as new molecular resources to regulate this process.

Firstly, marine-derived terpenoids were shown to play a significant role in regulating GPX4 activity. For example, scalarane-type sesquiterpenes isolated from sponges were found to induce elevated levels of lipid peroxidation in tumor cells by inhibiting GPX4 protein stability, thereby selectively triggering ferroptosis [30]. Specifically, Heteronemin(2) (see Figure 1B for chemical structure) in scalarane-type sesquiterpenes can induce the formation of ROS in tumor cells and trigger ferroptosis by inhibiting the expression of GPX4 [31,32]. The mechanism of scalarane-type sesquiterpenes triggering ferroptosis is shown in Figure 2. The unique isoprene backbone structure of this class of compounds may interfere with the enzymatic active center of GPX4 through hydrophobic interactions, and its mechanism of action is different from that of traditional small molecule inhibitors, providing new ideas for targeting GPX4 degradation [33]. Notably, some marine terpenoids can also coregulate iron metabolization-related proteins, such as TfR1 and FTH1, forming a multi-target intervention network [25].

At the level of lipid metabolism regulation, marine polyphenols exhibit unique dual regulatory properties. At the level of lipid metabolism regulation, fucoxanthin(3) (see Figure 1C for chemical structure) exhibits unique dual regulatory characteristics. It can both inhibit the expression of the key enzyme ACSL4(1) involved in lipid peroxidation and activate the Nrf2 signaling pathway, enhancing the transcriptional levels of GPX4, thereby reducing the occurrence of lipid peroxidation and protecting cells from oxidative damage [34,35]. The regulatory mechanism of fucoxanthin(3) on lipid metabolism is shown in Figure 2. This seemingly contradictory regulation mode actually forms a dynamic balance, which maintains normal cellular redox homeostasis while selectively killing tumor cells through microenvironment-dependent dose effects. Furthermore, bromophenolic compounds isolated from red algae were shown to specifically enhance the sensitivity of tumor cells to GPX4 inhibitors by hijacking the mitochondrial CoQ10 synthesis pathway [36]. The regulatory mechanism of brominated phenol compounds on the GPX4 pathway is shown in Figure 2. This mechanism of action breaks through the limitation of traditional ferroptosis inducers on cytoplasmic targets and reveals a new dimension of the subcellular metabolic network in the regulation of ferroptosis.

Ocean-specific sulfur-containing compounds exhibit irreplaceable chemical properties in the regulation of ferroptosis. Specifically, thioalkaloids biosynthesized by deep-sea hydrothermal vent organisms (such as ML210(4) (see Figure 1D for chemical structure) and its derivatives) can covalently modify the selenocysteine active site of GPX4, leading to an irreversible loss of its antioxidant function [37]. ML210(4) is a known covalent inhibitor of GPX4, which contains a key sulfur-containing group (such as thioacetate) in its structure. ML210(4) can covalently bond with the selenocysteine (Sec) residue of GPX4, forming a stable covalent bond [37,38]. The regulation of thioalkaloids on ferroptosis is shown in Figure 2. This unique sulfurotropic mechanism overcomes the targeting problem caused by the highly reduced environment of GPX4 protein and provides a new strategy for solving the selectivity problem of GPX4-targeted drugs. Meanwhile, some thioglycosides such as Bavachin(5) (see Figure 1E for chemical structure) can also enhance the autophagic degradation of ferritin while inhibiting cystine uptake by activating the p53-SLC7A11 (cystine/glutamate antiporter (system Xc^−^)) signaling axis, resulting in a synergistic effect of iron overload and antioxidant system inhibition [39,40,41]. Bavachin(5) specifically induces ferroptosis by inhibiting the phosphorylation of p-STAT3, activating P53, which in turn down-regulates the expression of SLC7A11, leading to GSH depletion and ROS and MDA accumulation [42].

Notably, marine sulfated polysaccharides (SPs) have opened up a new pathway for the regulation of ferroptosis. Fucoidan sulfate(6) (see Figure 1F for chemical structure) extracted from the sea cucumber body wall was shown to coordinate the regulation of key regulators such as GPX4, FSP1, and long-chain acyl-CoA synthetase 3 (ACSL3) by activating Farnesoid X receptor (FXR), independent of the traditional REDOX system [34]. This nuclear receptor-mediated epigenetic regulation mechanism makes it possible to overcome the adaptive resistance of tumor cells to single-target inhibitors. More strikingly, lipopeptides produced by some deep-sea bacteria can directly enhance the local accumulation efficiency of lipid peroxidation products at the plasma membrane by disrupting the asymmetric distribution of phospholipids [43]. This physico-chemical intervention overcomes the limitation of traditional ferroptosis inducers that rely on biological metabolic pathways.

Based on existing studies, we proposed that marine natural products have three unique advantages in the field of ferroptosis regulation. First, halogenated metabolites (e.g., bromotyrosine, chlorinated terpenes) produced by halogenated enzyme systems unique to marine organisms can improve their efficiency on membrane-bound targets (e.g., GPX4, ACSL4(1)) by enhancing molecular lipophilia. Second, rigid molecular skeletons (e.g., caged diterpenoids, helicoid alkaloids) are shaped by the high-pressure environment of the deep ocean. Thirdly, chimeric polyketide non-ribosomal peptides produced by commensal microorganisms may mimic the endogenous siderophore system to achieve specific regulation of iron metabolism. These characteristics suggest that we should establish an evaluation system for the ferroptosis activity of marine compounds, focusing on their effects on the redox microenvironment of subcellular membrane systems and the role of transmembrane transport properties in determining tumor selectivity. Future studies can combine single-cell lipidomics technology to analyze the spatial distribution characteristics of lipid peroxidation products induced by marine compounds, which will provide key theoretical support for the development of tissue-specific ferroptosis inducers.

## 2. Marine Compounds and Ferroptosis

### 2.1. Molecular Mechanisms of Marine Compounds Targeting Ferroptosis Pathways

In recent years, studies on the molecular mechanism of marine-derived compounds targeting ferroptosis pathway showed unique advantages in biological activity. First, marine-derived peptides such as Actin-derived small molecular peptide (ASMP(g), ASMP(7), see Figure 1G for chemical structure) were found to induce ferroptosis by interfering with the Nrf2/SLC7A11/GPX4 signaling axis [34,44]. Specifically, ASMP(7) inhibited the expression of SLC7A11, resulting in decreased intracellular cystine uptake and limited GSH synthesis, thereby weakening the ability of GPX4 to neutralize lipid peroxidation [34,45]. Notably, GPX4 is a core regulator of ferroptosis, and its loss of activity leads to the irreversible accumulation of lipid peroxidation products (such as malondialdehyde (MDA) and 4-Hydroxynonenal (4-HNE)) on the cell membrane, which eventually leads to membrane structure breakdown [46,47]. This mechanism was verified in a variety of tumor models. For example, in glioma cells, ASMP(7) significantly enhances the sensitivity of cells to lipid peroxidation by activating ACSL4(1) to promote the integration of PUFAs into membrane phospholipids [34].

**Figure 3:**
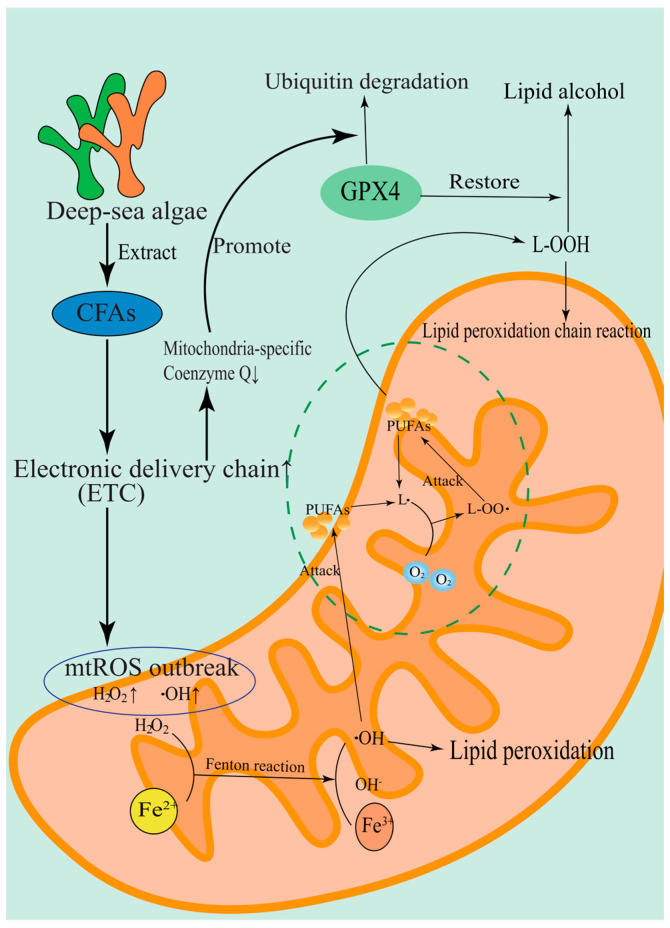
A schematic diagram of the mechanism of the effect of deep-sea algae extract on iron death-related lipid peroxidation and mitochondrial function. This figure illustrates how deep-sea algae extracts influence the process of ferroptosis by regulating lipid peroxidation and mitochondrial function. The key processes involved in the figure include lipid peroxidation chain reactions, the mitochondrial ETC, iron-catalyzed reactions, and the regulation of GPX4.

Cyclic fatty acids (CFAs), which are abundant in marine natural products, exhibit another unique pathway to regulate ferroptosis. It was found that CFAs derived from deep-sea algae can selectively target the mitochondrial membrane structure and promote mitochondrial reactive oxygen species’ (mtROS) explosion by enhancing the activity of the electron transport chain (ETC) [43]. This process is closely related to mitochondrial lipid peroxidation, which is the key execution link of ferroptosis. When the level of mitochondria-specific CoQ is depleted due to the excessive activation of ETC, the antioxidant defense system of the mitochondrial membrane collapses, leading to the ubiquitination and degradation of GPX4 protein, thereby magnify the ferroptosis effect [36]. The mechanism by which CFAs amplify ferroptosis effects is shown in Figure 3. The sensitivity of Rho0 cells (which lack functional ETC) to GPX4 inhibitors was more than 3-fold higher than that of wild-type cells, confirming the pivotal role of mitochondrial lipid REDOX homeostasis in the regulation of ferroptosis [36].

In terms of TME remodeling strategies, marine-derived nanocarrier technology provides a new idea for the regulation of lipid metabolism. For example, functionalized carbon dots loaded with copper ions (FG-CDs@Cu) can simultaneously consume excess GSH and release Cu^2+^ from TME, catalyzing the lipid peroxidation chain reaction via the Fenton reaction [48]. This dual effect not only directly induces ferroptosis of tumor cells but also activates dendritic cell maturation through the release of damage-related molecular patterns (DAMPs) and promotes anti-tumor immune responses [49,50]. In addition, the inactivation of GPX4 triggered by FG-CDs@Cu enhances the sensitivity of tumor cells to IFN-γ and TNF-α, forming a “positive ferroptosis-immunity cycle”, which reduced the tumor volume by 67% in a model of lung metastasis of melanoma [48].

From the perspective of lipid metabolism reprogramming, marine terpenoids such as sponge sesquiterpene lactone(8) (see Figure 1H for chemical structure) can specifically inhibit bromodomain-containing protein (BRD4), blocking its binding to the ACSL4(1) promoter region, thereby inhibiting the ACSL4(1)-mediated generation of lipid peroxide precursors [51]. This finding revealed a potential link between epigenetic regulation and ferroptosis, as BRD4 inhibitor treatment reduced the IC50 value of colorectal cancer cells to erastin (ferroptosis inducer) to one-fifth of the previous level, and the effect was more significant in cancer stem cell subsets with active lipid metabolism [51]. At the same time, some marine steroids can up-regulate the expression of GPX4, FSP1, and PPARα by activating FXR, forming a multi-layered lipid peroxidation defense network, which provides a potential target for reversing tumor ferroptosis resistance [52].

### 2.2. Clinical Translational Challenges of Targeting Ferroptosis Pathways with Marine Compounds

Although existing studies revealed that marine compounds induce ferroptosis through multiple pathways such as GPX4 regulation, mitochondrial targeting, and lipid metabolism intervention, there are still two bottlenecks in the clinical translation of marine compounds. First, although the lipophilic characteristics of marine natural products facilitate cell membrane penetration, they are easy to bind to serum proteins in TME, resulting in decreased bioavailability. In the future, it is necessary to combine with nano-encapsulation technology or a prodrug strategy to optimize the delivery efficiency. For example, cyclic fatty acids (CFAs) can promote ferroptosis through ETC activation in sarcoma but may have antagonistic effects due to FXR activation in liver cancer, suggesting the need to establish precise intervention programs based on tumor metabolic heterogeneity [43,52]. Notably, the unique pools of secondary metabolites shaped by extreme environmental stresses in marine ecosystems, such as lipopeptides derived from hyperbaric deep-sea microorganisms, may contain novel targets for ferroptosis regulation. Therefore, the systematic screening and mechanistic elucidation of such resources will be the key to a breakthrough in this field.

## 3. Mechanisms, Strategies, and Challenges of Marine Compounds in Regulating Ferroptosis

### 3.1. Core Signaling Pathways and Therapeutic Targets of Ferroptosis

#### 3.1.1. GPX4-Dependent Pathways

Ferroptosis can be activated by oxidative turbulence in the intracellular microenvironment, which is mainly controlled by GPX4 [7]. The GPX4-dependent pathway, with GPX4 as the core molecule in the ferroptosis regulatory network, plays a key role in maintaining cellular REDOX homeostasis through its antioxidant function. The enzymatic activity of GPX4 is dependent on the reducing ability of glutathione (GSH) and can reduce lipid peroxides (such as phospholipid hydroperoxides) in the cell membrane to non-toxic lipid alcohols, thereby blocking the diffusion of the lipid peroxidation chain reaction [53,54]. This mechanism protects cells from oxidative damage under physiological conditions, but in the TME, the overexpression of GPX4 may become a “protective umbrella” of cancer cells against ferroptosis, allowing them to survive under oxidative stress [54,55]. Notably, the molecular function of GPX4 is closely related to the selenocysteine residues in its structure, and this unique Se-dependent catalytic center makes it a “molecular switch” for the regulation of ferroptosis [22,56].

The inhibitor of GPX4, RSL3 (Generic GPX4 inhibitor, commonly used in ferroptosis research), directly inhibits the enzymatic function of GPX4 by covalently binding to its active site, leading to the accumulation of lipid peroxides and triggering ferroptosis [57,58]. Experimental studies showed that the GPX4 protein expression level was significantly decreased in tumor cells treated with RSL3, accompanied by mitochondrial membrane potential collapse, ROS surge, and iron ion metabolism imbalance, which all point to the typical phenotype of ferroptosis [57,59]. In addition, the effect of RSL3 is highly selective, and its mechanism of action is not dependent on traditional apoptosis or autophagy pathways but by disrupting the GPX4-mediated lipid repair system, making cells extremely sensitive to oxidative damage [22,60]. However, existing GPX4 inhibitors, such as RSL3 and ML162, face challenges in clinical translation, including off-target effects, insufficient metabolic stability, and potential toxicity to normal tissues [61,62,63]. This has prompted researchers to explore new regulatory strategies, especially to find compounds from natural products with GPX4 targeting potential.

Due to its unique chemical environment, marine ecosystems are rich in bioactive molecules, among which selenium compounds may regulate ferroptosis by mimicking or interfering with the seleno-dependent catalytic mechanism of GPX4. For example, some marine-derived selenomethionine(9) (see Figure 1I for chemical structure) analogues were shown to enhance the enzymatic activity of GPX4 and inhibit lipid peroxidation by up-regulating its protein expression or stabilizing its conformation [64,65]. In contrast, some marine polyphenols, such as SP from brown algae extracts, showed dual effects: indirectly promoting GPX4 expression at low concentrations by activating the Nrf2 pathway while inhibiting GPX4 function at high concentrations by chelating selenium elements or competitively binding to the GPX4 active site [34,66]. This dose-dependent regulatory property provides a new idea for the precise regulation of ferroptosis in tumor cells. More interestingly, certain marine natural products, such as sponge-derived sesquiterpenoids, are able to alter GPX4 subcellular localization or stability by inducing post-translational modifications, such as phosphorylation or ubiquitination, thereby disrupting the REDOX balance without completely inhibiting its activity [67,68]. This “mild regulation” mode may reduce the toxic side effects of traditional GPX4 inhibitors while maintaining selective pressure on tumor cells.

Based on the current research progress, we believe that marine compounds have unique advantages in the field of GPX4-targeting regulation. First, the antioxidant molecular pool evolved by marine organisms to adapt to high-salt, high-pressure, and low-light environments may contain GPX4 modulators that are distinct from those of terrestrial organisms. Second, selene-containing marine compounds, such as selenopolin analogues, can specifically bind to GPX4 through structural simulations, and their three-dimensional configuration and electron distribution may be superior to synthetic small molecules. Third, the synergistic effect of multiple active molecules in marine multicomponent extracts may enhance the ferroptosis induction efficiency through a multi-target action (such as simultaneous regulation of GPX4, SLC7A11, and ACSL4(1)) [34,69]. However, there are still many gaps in current research. For example, most of the regulatory mechanisms of GPX4 by marine compounds remain in the stage of phenotypic observation and lack mechanism verification by co-crystal structure or molecular dynamics simulation. Moreover, the bioavailability and transmembrane transport efficiency of marine-derived Se compounds have not been systematically evaluated. Future studies should combine artificial intelligence-assisted virtual screening technology to excavate lead compounds with GPX4 binding potential from the marine natural product library and optimize their pharmacokinetic properties through rational design. At the same time, the development of marine molecular chimeras based on GPX4 degradation-targeting chimeras technology may provide a breakthrough direction for overcoming the resistance of GPX4 inhibitors [70,71].

#### 3.1.2. ACSL4(1)-Driven Lipid Peroxidation

ACSL4(1)-driven lipid peroxidation ACSL4(1) (long-chain acyl-coa synthetase 4), as the core regulator of ferroptosis, plays a key role in tumor ferroptosis by catalyzing the binding of PUFAs to coenzyme A and driving the lipid peroxidation process of cell membrane phospholipids [72,73]. Notably, ACSL4(1) promotes the esterification of ω-6 PUFAs such as arachidonic acid (AA) and adrenal acid (AdA) to generate specific phospholipid substrates, which are highly susceptible to the formation of lipid peroxides (LPO) under iron-dependent oxidative stress conditions, ultimately leading to cell membrane rupture and ferroptosis [74]. The high expression of ACSL4(1) is significantly correlated with ferroptosis sensitivity in a variety of tumor models. For example, in glioma, ACSL4(1)-mediated enhanced lipid peroxidation can significantly enhance the anticancer effect [74]. In addition, the protein stability of ACSL4(1) is regulated by ubiquitination modification. For example, the UBR5-mediated ubiquitination degradation of ACSL4(1) can inhibit lipid peroxidation and reduce the sensitivity of colorectal cancer cells to ferroptosis [12].

In recent years, marine natural products have attracted much attention as potential sources of ACSL4(1) inhibitors. For example, sponge-derived dactylospene F, scalarin(10) (see Figure 1J for chemical structure), and honulactone A(11) (see Figure 1K for chemical structure) were confirmed to exhibit significant biological activity. These compounds effectively block the process of PUFAs’ esterification by targeting and inhibiting the catalytic activity of long-chain acyl-CoA synthetase 4 (ACSL4(1)). This mechanism is crucial for regulating lipid metabolism within cells, as the esterification of PUFAs is a precursor step in lipid peroxidation reactions. By inhibiting the activity of ACSL4(1), these sponge-derived sesquiterpene compounds can significantly reduce the accumulation of lipid peroxidation products, thereby alleviating oxidative stress damage at the cellular level [34]. Notably, this class of compounds showed dual effects in a nasopharyngeal carcinoma (NPC) model. On the one hand, it reduced the migration and invasion ability of tumor cells by inhibiting ACSL4(1), and on the other hand, it enhanced the imbalance of the oxidative stress defense system by regulating the Nrf2/SLC7A11/GPX4 signaling axis, thereby synergistically inducing ferroptosis [75]. In addition, marine-derived ASMP(7) (alginate sulfate polysaccharide) was also confirmed to promote lipid peroxidation by up-regulating ACSL4(1) expression, thereby activating the ferroptosis pathway. This mechanism provided a new idea for the development of ferroptosis inducers based on marine compounds [34].

Although the role of ACSL4(1) in ferroptosis was well established, the functional heterogeneity of ACSL4(1) in different tumor types still needs to be further explored. For example, in vascular endothelial cells, the loss of ACSL4(1) can significantly alleviate ferroptosis-related tissue damage by inhibiting lipid peroxidation, but in epithelial tumor cells, the high expression of ACSL4(1) instead serves as a marker of ferroptosis susceptibility [75,76]. This paradoxical phenomenon suggests that the regulation of ACSL4(1) may be tissue-specific, and its molecular mechanism may involve signal cross-talk in the microenvironment. In addition, the existing marine-derived ACSL4(1) inhibitors still face the challenges of insufficient selectivity and poor in vivo stability. Future studies can focus on optimizing the pharmacokinetic properties of the compounds through structural modifications or developing ACSL4(1)-targeting nano-delivery systems to improve its enrichment efficiency in tumor tissues [77]. Meanwhile, dual inhibition strategies combining ACSL4(1) with other ferroptosis-related targets, such as GPX4 or FSP1, may provide more effective therapeutic options to overcome tumor drug resistance [78].

#### 3.1.3. Iron Metabolism Network

The iron metabolism network plays a pivotal role in the regulation of ferroptosis in tumor cells. TfR1 is a key molecule of iron uptake, and its expression level is directly related to ferroptosis sensitivity. Studies showed that TfR1 significantly increases the intracellular labile iron pool (LIP) content by mediating the endocytosis of the transferrin/iron complex, which provides Fe^2+^ for the Fenton reaction, thereby promoting the peroxidation of PUFAs [78]. Notably, in tumor models where GPX4 activity is suppressed, the high expression of TfR1 accelerates the accumulation of lipid peroxidation products, which eventually leads to the irreversible disruption of the cell membrane integrity [79]. This regulatory mechanism was validated in a variety of solid tumors, such as in triple-negative breast cancer cell lines; the knockdown of TfR1 by RNA interference significantly reduced the efficiency of erastin-induced ferroptosis, accompanied by a decrease in the intracellular MDA level and the recovery of glutathione (GSH) content [80].

In this context, marine-derived iron chelators exhibit unique synergistic effects. Algal polyphenols have a strong iron-chelating ability due to their polyhydroxy structure and can interfere with the Tfr1-mediated iron uptake process by competitively binding Fe^3+^. Experimental evidence showed that brown algae polyphenols extracted from brown algae could dose-dependently reduce the expression level of TfR1 protein and inhibit the hepcidin signaling pathway in hepatocellular carcinoma HepG2 cells. This dual effect leads to the dysfunction of tumor cells in maintaining normal iron homeostasis [81]. Notably, these compounds, when combined with classical ferroptosis inducers such as RSL3, produced significant synergistic anti-tumor effects by reducing intracellular antioxidant reserves such as GSH and increasing the accumulation of lipid peroxidation products. In an animal model, the tumor volume of the combined treatment group was reduced by 58% compared with the monotherapy group and no significant liver and kidney function damage was observed [79].

From the perspective of metabolic network regulation, the relationship between TfR1 and ferroptosis is not limited to the iron supply level. Recent studies found that the expression level of TfR1 is negatively correlated with the activity of the mitochondrial respiratory chain complex II, suggesting that TfR1 may affect the ferroptosis threshold by regulating cellular energy metabolism [82]. In pancreatic cancer cells, inhibition of TfR1 leads to increased succinate dehydrogenase (SDH) activity, thereby increasing mitochondrial ROS production. This mechanism forms a spatially complementary oxidative damage pattern with plasma membrane lipid peroxidation [78]. This provides a theoretical basis for the development of combined therapeutic strategies for tumor metabolic heterogeneity, especially for tumor subtypes with high mitochondrial respiratory activity: targeting TfR1 may produce more precise therapeutic effects.

In the direction of the development of marine natural products, existing studies suggest that algal polyphenols may affect the expression of genes related to iron metabolism through epigenetic regulation. For example, bromophenols isolated such as rosmarinic acid(12) (see Figure 1L for chemical structure) and its liposomes from red algae can significantly enhance the demethylation effect of histone deacetylase (HDAC) inhibitors on the promoter region of TfR1, and this epigenetic remodeling makes tumor cells 3.2-fold more sensitive to ferroptosis [81]. This mechanism breaks through the limitation of traditional iron chelators that only act on iron ions themselves and provides innovative ideas for the development of a new generation of ferroptosis inducers with multi-target regulatory properties. In addition, the cross-reactivity of the extreme-adapted metabolites of marine organisms, such as the siderophore analogues synthesized by hydrothermal vents, with the mammalian iron transport system deserves further investigation.

In order to solve the above problems, we believe that it is necessary to establish a more accurate system for monitoring the dynamic transport of TfR1, especially the effect of its conformational change on the efficiency of iron uptake under the pH fluctuation in the TME. Secondly, there is an urgent need for breakthroughs in the bioavailability optimization of marine iron chelators, which can improve their tumor targeting through nanocarrier embedding technology or prodrug design. Finally, attention should be paid to the interaction between the iron metabolism network and immune checkpoint pathway, such as the potential effect of the TfR1-regulated intracellular iron level on Programmed Death-Ligand 1 (PD-L1) expression, which provides a new research direction for the synergistic application of ferroptosis inducers and immunotherapy. Through multidisciplinary innovation, marine compounds targeting the iron metabolism network are expected to become a breakthrough strategy to overcome tumor treatment resistance.

### 3.2. Selection and Mechanism of Marine Natural Products

#### 3.2.1. Marine Ferroptosis Inducers

As a treasure trove of natural products, marine ecosystems have shown unique potential in the screening of ferroptosis inducers in recent years. Secondary metabolites evolved by marine organisms to adapt to extreme environments often have novel chemical structures and special activities targeting the biofilm system, which provides an important breakthrough for the development of new ferroptosis regulators targeting the lipid peroxidation pathway.

In a study of terpenoids derived from coral symbionts, researchers found that specific diterpene derivatives can induce ferroptosis by regulating the stability of GPX4. Peach leaf coral glycosides (Aucubins(13), see Figure 1M for chemical structure) are compounds extracted from peach leaf corals that can significantly inhibit iron death induced by erastin and RSL-3. In erastin-induced HK-2 cells, Aucubins(13) or their aglycones can effectively inhibit the decrease in GSH levels and GPX-4 content, thereby exerting an inhibitory effect on iron death [83]. Notably, these compounds were able to activate the chaperone-mediated autophagy pathway and significantly promote the lysosomal degradation of GPX4 [84]. This mechanism of action is complementary to the traditional GPX4 enzyme activity inhibition strategy and provides a new idea for solving the adaptive drug resistance of tumor cells to GPX4 inhibitors. Further studies showed that the degradation of GPX4 induced by terpenoids is tissue selective, and the degradation efficiency in tumor cells is significantly higher than that in normal cells, which may be related to the abnormal active protein degradation system in tumor cells. Interestingly, some coralline-derived terpenoids could synergistically enhance ACSL4(1)-mediated polyunsaturated fatty acid peroxidation, resulting in a dual mechanism of GPX4 degradation and lipid peroxidation [85].

Deep-sea sponge-derived polyketide compounds showed specific inhibition of ACSL4(1) activity. Plocabulin(14) (see Figure 1N for chemical structure) is a polyketide compound isolated from the sponge Lithoplocamia lithistoides. Our data showed that these polykekeys competitively bind to the substrate recognition domain of ACSL4(1) and inhibit the conversion of arachidonic acid to Acyl-CoA ester [86]. This inhibition directly reduces the proportion of polyunsaturated fatty acids in the cell membrane and reduces the material basis for lipid peroxidation from the source. Compared with the gene silencing of ACSL4(1), polyketo inhibitors retain the physiological function of ACSL4(1) in normal lipid metabolism and show better safety profile [87]. More interestingly, some polyketo molecules can induce a conformational change in ACSL4(1) to expose new ubiquitination sites, thereby promoting the proteasomal degradation of ACSL4(1). This dual mode of “inhibitor-degradation” provides a chemical basis for the development of long-acting ACSL4(1) modulators [88].

From the perspective of drug development, the unique value of marine-derived ferroptosis inducers lies in their multi-target regulatory properties. For example, some terpene-polyketo hybrids can affect GPX4 stability and ACSL4(1) activity at the same time, and this synergistic effect can break through the efficacy bottleneck of single-target inhibition [87]. At the level of translational medicine, it is necessary to improve the metabolic stability of marine compounds and enhance their oral bioavailability through structural modification, while retaining their unique transmembrane permeability [89]. It is worth exploring that the special chemical structures shaped by the extreme marine environment may give these compounds unique subcellular targeting properties, such as specific enrichment in the mitochondrial membrane system and regulation of ferroptosis-related lipid metabolism, which provide natural templates for the development of precision delivery systems [36].

#### 3.2.2. Marine-Derived Ferroptosis Inhibitors

Polyphenols extracted from marine brown algae exhibit unique molecular mechanisms for regulating ferroptosis. Firstly, brown algae, the main marine source of polyphenols, exhibited significantly higher antioxidant activity than red and green algae [90]. The chemical structure of these polyphenols is rich in phenolic hydroxyl groups, which make them potent free radical trappers, especially against lipid free radical scavenging, the core driver of ferroptosis [91]. Notably, the molecular characteristics of brown algae polyphenols enable them to penetrate the cell membrane and directly neutralize alkoxide and peroxyl radicals generated by the lipid peroxidation chain reaction in the lipid bilayer, thereby blocking the execution of ferroptosis [92].

At the molecular mechanistic level, brown algae polyphenols not only act by physically trapping free radicals but also activate the antioxidant defense system in the cells. For example, DiEckol(15) (see Figure 1 for chemical structure), a characteristic brown algae polyphenol isolated from brown algae, was found to significantly reduce reactive oxygen species’ levels while inhibiting iron accumulation and the production of the lipid peroxidation marker MDA [93]. This dual mechanism of action allows it to intervene both in the initiation phase of ferroptosis (disturbance of iron metabolism) and in blocking the execution phase of ferroptosis (lipid peroxidation cascade) [94]. Notably, this protective effect is particularly pronounced in normal cells, while the ferroptosis process in cancer cells shows a feature of selective regulation, which is closely related to its ability to maintain redox homeostasis more efficiently in normal cells [95].

From the perspective of signal pathway analysis, brown algae polyphenols have regulatory effects on GPX4 signaling axis. Recent studies found that brown algae polyphenols’ extracts can enhance the activity of GPX4, and this activation may be achieved by stabilizing the GSH synthesis pathway [96]. Notably, this mechanism is different from the traditional GPX4-dependent pathway. Brown algae polyphenols can also maintain the redox balance of the cell membrane through non-GPX4 pathways, such as enhancing the antioxidant reserve of cells by up-regulating the expression of Nrf2 [27]. This multi-target action feature gives them unique advantages in dealing with the complex regulatory network of ferroptosis.

At the translational medicine level, the protective mechanism of brown algae polyphenols shows dose-dependent characteristics. Experimental data showed that the selective protective effect of brown algae polyphenols on normal cells could reach 82% when the concentration of brown algae polyphenols reached 50 μM, while the protective effect on tumor cells was only 17%. This selective protective effect may be due to the disorder of iron metabolism and the defect [97]. This selective protective effect may be due to the unique iron metabolism disorder and antioxidant system defects of tumor cells. When combined with the classical ferroptosis inhibitor Ferrostatin-1, brown algae polyphenols can increase the survival rate of normal cells to 95%, without affecting the killing effect of ferroptosis inducers on tumor cells [98].

It is worth further study that the protective effect of brown algae polyphenols is space–time specific. In the early stage of ferroptosis (0–6 h), it mainly acts through the direct scavenging of lipid free radicals, while in the later stage (6–24 h), it turns to regulate the expression of iron metabolism-related proteins [99]. This dynamic regulatory mechanism enables it to both rapidly neutralize acute oxidative stress and maintain long-term REDOX homeostasis. From the structure–activity relationship analysis, the pyrogalinol unit in brown algae polyphenols is crucial for its free radical-trapping ability, and the three adjacent hydroxyl groups in the structure can form a stable semiquinone radical intermediate, thereby efficiently terminating the lipid peroxidation chain reaction [100].

Based on the analysis of current research progress, we concluded that there are two key challenges for the clinical application of brown algae polyphenols as ferroptosis inhibitors. First, its transmembrane transport efficiency needs to be improved, which can be improved by nanocarrier technology. Second, a more precise delivery system needs to be established to achieve the specific protection of normal cells. Notably, the polyphenol structure of brown algae polyphenols enables them to naturally target the oxidative stress microenvironment, which can be exploited to design intelligent responsive drug delivery systems. In addition, considering the dual role of ferroptosis in cancer treatment, future studies should focus on exploring the time-window administration strategy of brown algae polyphenols, that is, to administer them before radiotherapy and chemotherapy to protect normal tissues and to suspend the administration during the treatment interval to maintain the ferroptosis sensitivity of tumor cells. This dynamic regulation strategy may open up a new path for targeted ferroptosis intervention in cancer therapy.

#### 3.2.3. Technology-Driven Screening Strategies

In the quest for marine-derived compounds to regulate tumor ferroptosis, a technology-driven screening strategy provided key methodological support for the discovery of lead molecules with lipid peroxidation and GPX4 signaling regulatory activities. The core of this strategy is to integrate the lipidomics analysis of targeted lipid peroxidation biomarkers and CRISPR-Cas9 gene editing technology to form a closed-loop research system from compound screening to target verification. Its scientific logic and technical path can be divided into the following three levels.

Firstly, high-throughput screening based on lipidomics provides an accurate molecular phenotypic analysis tool for the activity evaluation of marine compound libraries. Lipid peroxidation is the core execution mechanism of ferroptosis, and the dynamic changes in its biomarkers (such as phospholipid hydroperoxides, malondialdehyde, etc.) can directly reflect the degree of ferroptosis. Targeted lipidomics enables the whole-body lipid profiling of tumor cells treated with marine compounds, focusing on the types and contents of oxidized phospholipids associated with PUFAs, to screen candidate molecules [101]. For example, the depletion of mitochondria-specific CoQ was shown to significantly promote GPX4 inhibition induced ferroptosis by enhancing mitochondrial lipid peroxidation, a finding that suggests that marine compounds may regulate ferroptosis sensitivity by targeting mitochondrial lipid metabolism pathways [43]. In addition, lipidomics data can be integrated with transcriptome or metabolome data to reveal the remodeling characteristics [102,103].

Notably, CRISPR-Cas9 genome-wide screening technology has played an irreplaceable role in target verification and mechanism elucidation. By constructing genome-wide CRISPR knockout libraries, researchers can systematically screen for genetically dependent genes related to ferroptosis induced by marine compounds. For example, a study on ovarian cancer using CRISPR-Cas9 screening showed that deletion of the transcription factor PAX8 significantly enhanced the pro-ferroptosis effect of the GPX4 inhibitor RSL3, revealing PAX8 as a novel regulator of GPX4-dependent ferroptosis resistance in tumor cells [104]. Similarly, the CRISPR screening strategy based on flow cytometry sorting successfully identified mucosa-associated lymphoid tissue lymphoma translocation protein 1 (MALT1) to affect the ferroptosis process by regulating GPX4 stability, providing a mechanistic validation framework for marine compounds targeting the GPX4 degradation pathway [105]. Such techniques can not only validate the functions of known ferroptosis-related genes (such as GPX4 and ACSL4(1)) but also discover new regulatory factors. For example, CRISPR screening can be used to identify the key deubiquitinizing enzymes’ (DUBs) targets under the treatment of ferroptosis inducers, which lays a foundation for an in-depth analysis of the mechanism of marine compounds [106].

Furthermore, the combination of lipidomics and CRISPR-Cas9 can be used to construct a “phenotype–genotype” dual-dimensional verification system. Taking the phenotype of lipid peroxidation induced by marine compounds as a starting point, researchers can screen the gene clusters significantly related to this phenotype through the CRISPR library and then analyze the changes in lipid metabolites after these gene knockouts using lipidomics to reveal the regulatory network of the compound–target–lipid metabolism axis. For example, GPX4 deletion results in the extreme sensitivity of cells to mitochondrial lipid peroxidation, suggesting that marine compounds may trigger ferroptosis by mimicking GPX4 repression, and CRISP-mediated GPX4 knockout experiments can verify whether candidate compounds exert their effects by directly or indirectly inhibiting GPX4 [36]. In addition, CRISPR screening data of iron metabolic-related genes (such as TfR1 and ferritin) can help to determine the iron-dependent effects of marine compounds and avoid the misjudgment of non-specific oxidative stress effects of ferroptosis [78].

From the perspective of translational medicine, the innovation of this technical strategy lies in its ability to break through the limitations of traditional drug screening. Existing ferroptosis inducers, such as RSL3 and erastin, often have limited efficacy in animal models due to poor metabolic stability or insufficient targeting. Marine natural products may provide more selective ferroptosis regulatory molecules due to their structural diversity and biocompatibility advantages [28]. For example, mitochondria-targeting fatty acids were shown to inhibit tumor growth by inducing mitochondrial lipid peroxidation and GPX4 degradation, which provides a theoretical basis for the design of marine-derived mitochondria-targeting ferroptosis inducers [43]. At the same time, CRISPR technology can assist in optimizing a compound structure–activity relationship (SAR), such as validating the specificity of candidate compounds by knocking out their putative target genes, thereby reducing the risk of off-target effects.

Although existing technologies have enabled high-throughput screening, the molecular mechanisms by which marine compounds regulate ferroptosis often involve the synergistic action of multiple targets and pathways. By integrating the lipidome, transcriptome, and CRISPR screening data, machine learning algorithms can be introduced to predict the targets and pathway networks of compounds more efficiently. For example, the ferroptosis sensitivity prediction model is constructed based on the GPX4 regulatory network [55].

### 3.3. Remodeling Strategies of TME

#### 3.3.1. Reprogramming of Lipid Metabolism

Tumor cells establish resistance to ferroptosis through lipid metabolic reprogramming, in which the dynamic balance of lipid droplets and the regulation of PUFA metabolism are the key links. Lipid droplets, as lipid storage organelles, significantly reduce the abundance of lipid peroxidation substrates in cell membrane phospholipids by sequester-ing peroxidation-sensitive polyunsaturated fatty acids (such as arachidonic acid and adrenal acid), thereby forming a buffer barrier against ferroptosis [107]. Notably, the abnormal accumulation of lipid droplets in aggressive tumors such as glioblastoma was confirmed to be closely related to ferroptosis tolerance. This metabolic adaptation is not only dependent on the activity of lipid synthase (such as ACSL4(1) and LPCAT3) but also on the inhibition of lipid droplet catabolic pathways (such as autophagy-dependent lipid droplet degradation) [107]. In this context, marine-derived compounds showed unique intervention potential, such as Fucoidan and fucoxanthin(3), which can reduce de novo fatty acid synthesis and limit lipid droplet biogenesis by inhibiting the sterol regulatory element binding protein (SREBP) signaling pathway, thereby breaking the defense mechanism of ferroptosis in tumor cells [108].

**Figure 4:**
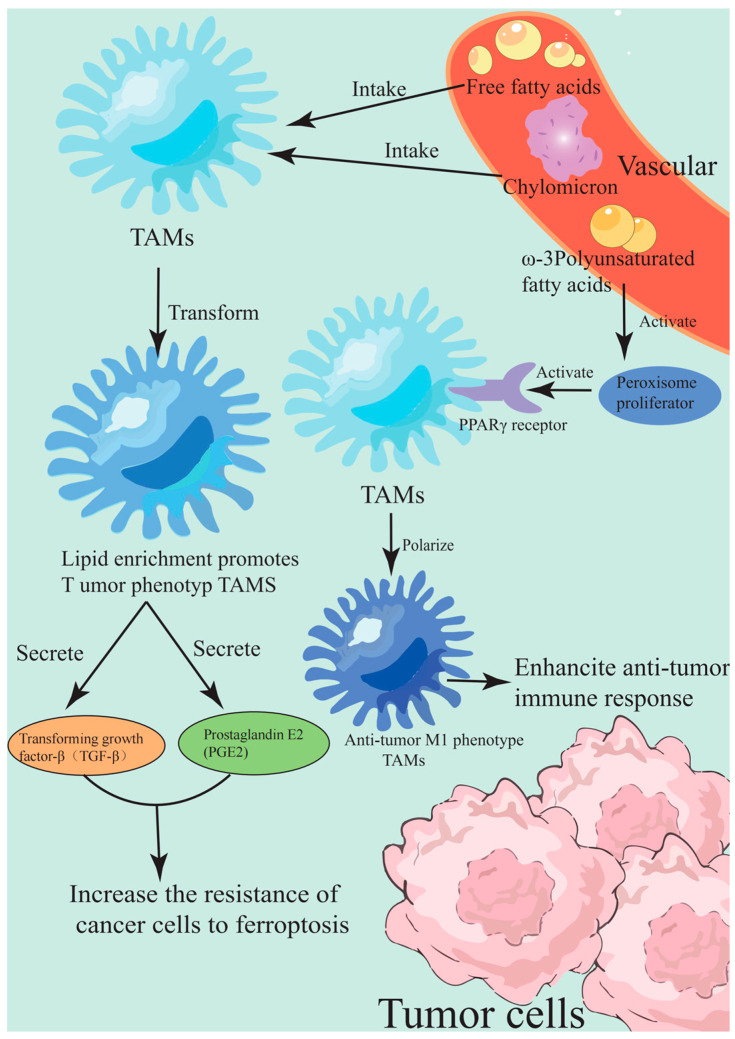
The polarization of TAMs and its mechanism affecting the ferroptosis resistance of tumor cells. This figure illustrates the polarization process of TAMs under the influence of lipid enrichment and PUFAs as well as its impact on the ferroptosis resistance of tumor cells.

Another key target of lipid metabolic reprogramming is the metabolic phenotype regulation of TAMs. TAMs form a lipid-enriched pro-tumor phenotype by ingesting chylomicrons’ remnants and free fatty acids in the TME. This metabolic reprogramming not only supports their immunosuppressive function but also enhances the ferroptosis resistance of adjacent cancer cells by secreting mediators such as transforming growth factor-β (TGF-β) and prostaglandin E2 (PGE2) [109]. Marine-derived omega-3 polyunsaturated fatty acids (e.g., eicosapentaenoic acid (EPA) and docosahexaenoic acid (DHA)) and their oxidative derivatives (e.g., resolvin E1) have dual regulatory effects. On the one hand, they reduce the production of pro-inflammatory mediators by the competitive inhibition of arachidonic acid metabolism, and on the other hand, they promote the polarization of TAMs to an anti-tumor M1 phenotype by activating peroxisome proliferator-activated receptor γ (PPARγ) [110]. The regulatory mechanism of TAMs on the metabolic phenotype reprogramming of lipid metabolism is shown in Figure 4. In the HCC model, DHA treatment significantly reduced the lipid accumulation of TAMs and restored their antigen presentation function. At the same time, DHA treatment increased the sensitivity of tumor cells to erastin-induced ferroptosis by more than 3-fold by up-regulating the expression of GPX4 inhibitor ACSL4(1) [111].

Notably, the molecular association between lipid droplets and ferroptosis resistance was spatio-temporally specific. In metastatic cancer cells, lipid droplets maintain cell survival by sequestering free iron and lipid peroxidation byproducts, whereas marine terpenoids such as sponge-derived Manzamine A(16) (see Figure 1P for chemical structure) specifically interfere with lipid droplet–lysosome interactions, promoting the release and reintegration of stored PUFA into cell membrane phospholipids. This metabolic redistribution renders metastatic cancer cells more susceptible to ferroptosis under oxidative stress to the circulatory system [112]. In line with this, a model of brain metastasis from breast cancer showed that neuroprotectin D1, a DHA derivative, can accelerate lipid droplets’ breakdown and release ω-6 PUFA by activating adipose triglyceride lipase (ATGL), leading to an imbalance of the ω-6/ω-3 ratio in the lipid composition of metastatic cell membrane. In turn, it initiates a lethal lipid peroxidation cascade [51].

Recent studies found that exosomes secreted by TAMs can deliver miR-27a-3p to cancer cells to limit exogenous lipid uptake by inhibiting the expression of fatty acid transporter CD36, forcing cancer cells to rely on the GPX4 pathway to maintain REDOX homeostasis, which provides a new idea for the combined treatment of marine compounds [113]. For example, didemnin B derived from sea squirt can achieve a synergistic effect of ferroptosis induction and immune microenvironment remodeling in a pancreatic cancer model by blocking eukaryotic translation initiation factor (eIF) activity and inhibiting GPX4 protein synthesis and lipid uptake-related genes’ (such as FABP4) expression of TAMs [114].

Existing studies confirmed that the mechanism by which marine compounds enhance ferroptosis sensitivity through lipid metabolic reprogramming is tissue-specific. For example, it mainly affects the fatty acid synthesis pathway in glioma, while it more affects lipid droplet homeostasis in breast cancer. This difference suggests that future drug development needs to take into account the metabolic heterogeneity of tumors to design precise intervention strategies. It is worth noticing that most studies focused on the regulation of PUFA metabolism but ignored the regulation of ferroptosis by sphingolipid metabolism (such as ceramide synthetase). Marine-derived sphingosine analogues (such as agelasphin isolated from red algae) may become a new breakthrough. In addition, the temporal and spatial effects of TAMs’ intervention in lipid metabolism are not clear—inhibition of TAMs’ lipid accumulation in the early tumor stage may enhance anti-tumor immunity, but excessive depletion of TAMs’ lipid reserves in the late stage may trigger a compensatory inflammatory response, which requires multi-omics analysis to reveal the key metabolic checkpoints. Based on the unique chemical diversity and targeting properties of marine compounds, the development of lipid droplet inhibitors that can penetrate the blood–brain barrier (such as modified squalene oxide) may break through the bottleneck of ferroptosis treatment in brain tumors [115].

#### 3.3.2. Oxidative Stress and Immune Regulation

**Figure 5:**
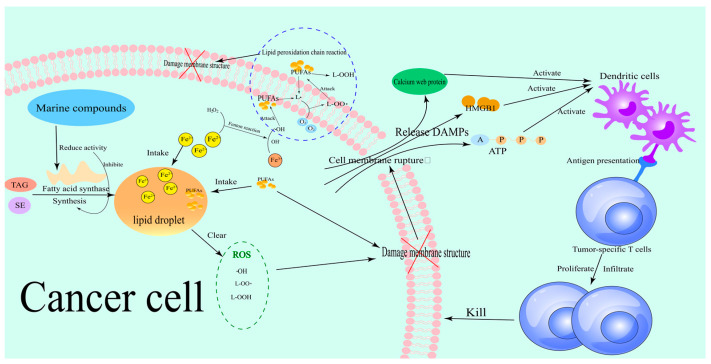
The interaction mechanism between lipid peroxidation and the tumor immune microenvironment. This figure illustrates the mechanisms of lipid peroxidation chain reactions and the resulting cellular damage, antigen presentation, and activation of tumor-specific T cells. Key processes involved in the figure include lipid peroxidation, cell membrane rupture, the release of DAMPs, and the activation of immune cells.

Ferroptosis-induced DAMPs’ release is one of the key mechanisms to activate anti-tumor immunity [116]. When tumor cells undergo ferroptosis, the accumulation of intracellular lipid peroxidation products and oxidative stress signals will lead to the rupture of the cell membrane and the release of a large number of immunogenic molecules, such as calreticulin, high mobility group box 1 (HMGB1), and ATP. These DAMPs activate the antigen-presenting function of dendritic cells and promote the proliferation and infiltration of tumor-specific T cells, thereby reshaping the immunosuppressive state of TME [117,118]. It is worth noting that marine-derived natural compounds exhibit unique regulatory capabilities in this process. For example, marine active compounds ASMP(7) significantly enhanced the accumulation of ROS and lipid peroxidation levels in cancer cells by inhibiting the Nrf2/SLC7A11/GPX4 antioxidant axis while up-regulating the expression of ACSL4(1), resulting in a dual ferroptosis-inducing effect [34]. The mechanism of DAMP activation of anti-tumor immunity induced by ferroptosis is shown in Figure 5. This effect not only directly kills tumor cells but also activates innate and adaptive immune responses through the release of DAMPs, providing a molecular basis for the synergistic effect of immune checkpoint inhibitors.

Recent studies found that the synergistic effect of ferroptosis induction and immune checkpoint blockade therapy is highly tumor-specific. Traditional ferroptosis inducers such as erastin or RSL3, while inhibiting GPX4 activity, often have toxicity to immune cells (such as T cells and NK cells), limiting their clinical transformation potential [119]. However, the marine-derived small-molecule compound N6F11 showed a breakthrough advantage in selectively inducing ferroptosis in cancer cells. By targeting the TRIM25-mediated GPX4 degradation pathway, N6F11 significantly enhanced the infiltration and cytotoxicity of CD8+ T cells in the TME while preserving immune cell function [119]. This selective mechanism provides a new idea for the development of low-toxicity ferroptosis-immune combination therapy.

In the cross field of oxidative stress and immune regulation, the multi-target characteristics of marine compounds are further highlighted. Some marine polyphenols, for example, Eckol(17) (see Figure 1Q for chemical structure), can simultaneously inhibit GPX4 activity in tumor cells and activate the stimulator of interferon genes (STING) signaling pathway in dendritic cells, thereby inducing ferroptosis and enhancing type I interferon secretion, which forms a positive immune regulatory loop [120]. In addition, the application of nano-delivery systems provides technical support for the synergy between marine compounds and immune checkpoint inhibitors. Some studies found that nanodrugs achieve the spatio-temporal synergy of ferroptosis induction and immune checkpoint blockade by loading marine active components and anti-PD-L1 antibodies in the local tumor, which not only significantly improves the activation level of T cells but also inhibits the growth of distant metastases [117]. The core of this strategy is to achieve the establishment of an anti-tumor immune memory under the premise of avoiding the excessive activation of systemic immunity by precisely regulating the level of lipid peroxidation.

From the perspective of existing research progress, the unique advantages of marine-derived compounds in the regulation of ferroptosis are mainly reflected in three aspects. First, the diversity of their chemical structures provides a rich molecular library for the development of selective modulators targeting key pathways such as GPX4/SLC7A11. Second, the multi-target nature of natural products helps to overcome the resistance of tumor cells to single ferroptosis inducers. Finally, the unique evolutionary pressures of marine ecosystems endow these compounds with a better immunomodulatory balance. However, the following challenges still need to be addressed. First, how to accurately screen ferroptosis inducers with synergistic immune effects to avoid functional inhibition of tumor-infiltrating lymphocytes? Second, we need to establish a better dynamic monitoring system for lipid peroxidation to evaluate the effects of different oxidative stress levels on immune cell subsets. Third, breakthroughs are still needed in the optimization of pharmacokinetics of marine compounds, especially for blood–brain barrier penetration and targeted delivery of tumors. Future research should focus on the construction of a “ferrodeath-immunomodulatory dual-function molecular library” and analyze the spatio-temporal transmission of oxidative stress signals in the TME through organoid models and single-cell sequencing technology, which will provide a theoretical basis for the design of personalized combination therapy.

## 4. Challenges and Solutions

### 4.1. Improve Bioavailability

To address the bioavailability bottleneck of marine-derived polyphenols in regulating ferroptosis, liposome-based nano-delivery systems provide an innovative technical path to solve this problem. Marine polyphenols are natural antioxidants and GPX4 signal conditioning factors. They have anti-tumor effects because of low solubility in water, the distribution of the instability of gastrointestinal tract, and are non-specifically severely limited. Because of their amphiphilic liposome bilayer structure, effective package loading of hydrophobic polyphenol, and ocean-form stable nanometer compound, this feature was verified in the resveratrol liposome research: after the encapsulation of the liposome drug particle size control at 333 nm, the coating rate reached 75.6%. The photostability and cellular uptake efficiency of the compound were significantly improved [121]. Notably, liposome surface modification technology can give it an active targeting ability, for example, by integrating transferrin receptor ligands, the drug delivery system can specifically recognize the overexpression of transferrin receptors on the surface of tumor cells, so as to achieve precise drug accumulation in the TME [122].

At the mechanistic level of improving bioavailability, the protective effect of liposomes on marine polyphenols is reflected in multiple dimensions. Firstly, the phospholipid layer shields the polyphenol molecules from digestive enzymes and reduces chemical degradation in the gastrointestinal tract. The retention rate of encapsulated shrimp lipid extract and fish oil in the simulated digestion environment was increased by more than 40% [123]. Second, nanoscale liposomes can enhance the penetration and retention effects through the enhanced permeability and retention (EPR)-mediated passive targeting of tumor tissues, promote cell membrane fusion, and simultaneously regulate surface charges to directly boost intracellular drug concentration. The biomimetic liposome system for macrophage membrane developed for naringenin achieved efficient crossing of the blood–brain barrier by mimicking the biological characteristics of natural cells, which is particularly important for the regulation of ferroptosis in central nervous system tumors [124].

It was proven that liposome delivery systems can increase the bioavailability of marine polyphenols by 3–5 times, which is not only due to physical protection but also closely related to the optimization of drug release kinetics. Multi-layer liposomes coated with marine polysaccharides (e.g., SP) and chitosan can be used to construct pH-responsive controlled release systems, which can trigger a burst release of drugs in the slightly acidic tumor environment while maintaining stability in the circulatory system [125]. This intelligent release property was confirmed by in vitro antioxidant activity experiments. The RSH-RSF coated with bilayer NPS achieved 85.26% and 80.48% activity retention rates in ABTS and DPPH free radical scavenging experiments, respectively [126].

Despite the progress made in the current research of marine polyphenol liposomes, two key challenges remain. First, the structure–activity relationships of most marine active ingredients have not been fully elucidated, resulting in a lack of rational guidance for the formulation of liposomes. For example, the interaction mechanism between echinochrome and red algae polysaccharide still needs to be further explored [127]. Secondly, how to maintain the physical stability and batch consistency of nanoparticles in the large-scale production process requires the development of novel microfluidic preparation technology and a freeze-drying protective agent system. Based on the above challenges, it is recommended that future research should focus on establishing a molecular database of marine polyphenols, combining artificial intelligence to predict their liposome compatibility parameters and developing hybrid liposomes with multiple responses to the TME, such as integrating photothermal conversion materials to achieve a coordinated regulation of drug release and treatment monitoring, which may open up a new technical paradigm for ferroptosis-targeted therapy.

### 4.2. Mechanisms of Drug Resistance

FSP1 is a novel factor of ferroptosis resistance (independent of the cysteine/GSH/ GPX4 axis), which reduces coenzyme Q10 expression, thereby leading to lipid peroxides’ accumulation [128]. The abnormal activation of the FSP1-CoQ10 pathway has become one of the key drug resistance mechanisms in tumor cells during anti-ferroptosis induction therapy. FSP1 forms a second defense barrier independent of the GPX4 system through its CoQ10-dependent antioxidant pathway, which significantly weakens the therapeutic effect of targeted lipid peroxidation strategies [129]. The core of this pathway is that FSP1 uses NAD(P)H to reduce oxidized CoQ10 to the free radical-trapping panneol form (CoQ10H2), thereby directly scavenging lipid peroxides and interrupting the ferrodeath cascade [130]. Of note, tumor cells can up-regulate FSP1 expression through epigenetic regulation or the aberrant activation of transcription factors such as Nrf2. In particular, in Kelch-like ECH-associated protein 1 (KEAP1) mutant non-small cell lung cancer (NSCLC), the overexpression of FSP1 induced cross-resistance to GPX4 inhibitors [131].

Recent studies found that persistent activation of the mitogen-activated protein kinase (MAPK)/Nrf2 signaling pathway in KRAS mutant tumors specifically induced high expression of FSP1, and this adaptive response enabled tumor cells to maintain REDOX homeostasis through the FSP1–CoQ10 axis after GPX4 inactivation [132]. More importantly, the membrane localization of FSP1 allowed it to directly neutralize lipid peroxides on the cell membrane, which explains why some tumors exhibit innate resistance to membrane-targeted ferroptosis-inducing agents [133]. Experimental evidence showed that in ALL, FSP1 promoter hypomethylation led to a high expression of the protein, which makes tumor cells resistant to glutathione system-targeted therapy. When FSP1 is inhibited simultaneously, the resistant phenotype can be significantly reversed [29].

**Figure 6:**
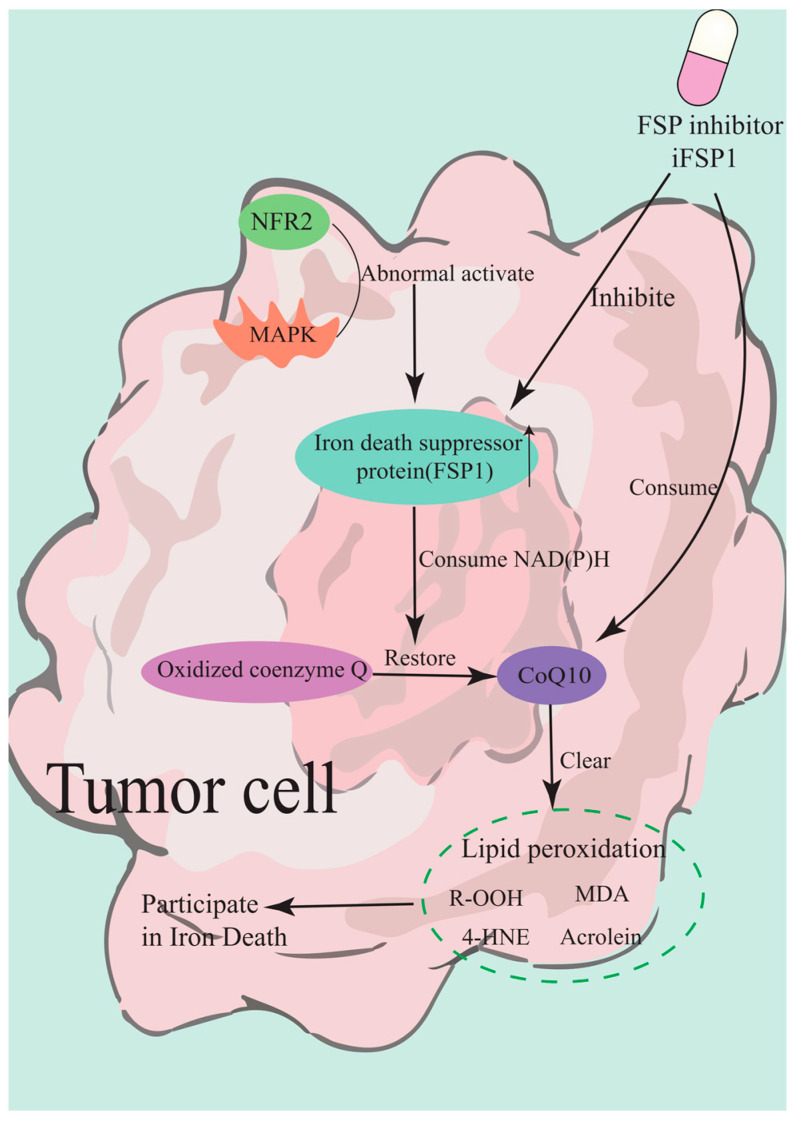
The regulatory mechanism and metabolic pathways of FSP1 in tumor cells. The figure illustrates the regulatory mechanism of FSP1 in tumor cells and its metabolic pathways. It reveals how FSP1 inhibits lipid peroxidation by consuming NAD(P)H and reducing oxidized coenzyme Q, thereby protecting cells from ferroptosis. Additionally, it demonstrates the regulatory role of the Nrf2 signaling pathway on FSP1 expression, as well as the potential toxic effects of lipid peroxidation products (such as MDA, 4-HNE, and acrolein) on cells.

In view of this resistance mechanism, researchers developed a variety of strategies to break the resistance. Gene-editing techniques confirmed that the combined silencing of FSP1 and Nrf2 produces a synergistic effect to reacquire ferroptosis sensitivity in KEAP1-mutated tumors [131]. In terms of drug development, the novel FSP1 inhibitor of FSP1 (iFSP1) combined with sonodynamic therapy can break through the antioxidant barrier of the cell membrane and overcome drug resistance by depleting CoQ10 reserves and enhancing lipid peroxidation accumulation [133]. The mechanism by which the novel FSP1 inhibitor iFSP1 overcomes the antioxidant barrier of the cell membrane is shown in Figure 6. It is worth noting that the targeted delivery system based on nanotechnology shows unique advantages. The neuron-targeted liposomal CoQ10 successfully restores the antioxidant function of FSP1 system in the blood–brain barrier injury model, which provides important implications for the targeted therapy of solid tumors [134].

However, there are still several key bottlenecks in the existing research. Firstly, the dynamic changes in NAD(P)H in the TME directly affect the activity of the FSP1–CoQ10 axis. However, real-time monitoring methods for this metabolic network have not been established. Secondly, the biodistribution of CoQ10 in the human body has not been fully simulated in preclinical models, especially its dynamic balance between mitochondrial membrane and plasma membrane, which may affect the evaluation of drug efficacy. Based on the above problems, future research should focus on the development of dual-targeting inhibitors that can penetrate the cell membrane, and at the same time integrate metabolomics technology to establish a dynamic drug resistance early warning system. New directions worth exploring include the use of AI algorithms to predict the dynamic structural changes in FSP1 to design allosteric inhibitors and the development of smart nanocatellors that can respond to oxidative stress in the TME to realize the spatio-temporal synergistic effect of CoQ10 depletion and FSP1 inhibition. These innovative strategies may break through the current treatment bottleneck and provide new solutions to overcome ferroptosis resistance.

### 4.3. Clinical Translation

In the process of clinical transformation of ferroptosis-targeted therapy, patient-derived organoids’ (PDO) platform, as a core tool for personalized drug screening, provides a key breakthrough to solve tumor heterogeneity and treatment resistance. Firstly, by preserving the genetic characteristics and phenotypic heterogeneity of the primary tumor, the PDO model can highly simulate the dynamic response of tumor to ferroptosis inducers in patients. For example, in the PDO model of BRCA1-deficient ovarian cancer, the combination of GPX4 inhibitors and PARP inhibitors can significantly enhance the synergistic anti-tumor effect induced by ferroptosis, which not only validates the reliability of PDO in predicting drug sensitivity but also provides an experimental basis for screening individualized treatment options for patients with specific genetic backgrounds [135]. Of note, the colorectal cancer PDO screening study further revealed that tumors with defective mitophagy are highly sensitive to ferroptosis-induced therapies, indicating that the PDO platform can accurately identify potential benefit populations by integrating molecular typing and functional validation [136].

However, the clinical translation of PDO still faces multiple challenges. On the one hand, the high complexity of the regulatory mechanism of ferroptosis leads to differences in the responses between its in vitro model and in vivo microenvironment. For example, the characteristics of the TME such as hypoxia and overexpression of glutathione may weaken the predictive value of ferroptosis inducers in PDO, which requires the integration of stromal cells and immune cells through a three-dimensional co-culture system to improve the biomimetic model [137]. On the other hand, as the core regulatory target of ferroptosis, GPX4 expression level and post-translational modification show significant heterogeneity in different PDO models, which may affect the stability [138]. To solve this bottleneck, the latest research proposes to combine CRISPR/Cas9 gene editing technology with the PDO platform to construct a GPX4 conditional knockout model, which can systematically evaluate the regulatory effect of targeted interventions on downstream molecules of the pathway (such as SLC7A11 and TFAP2A), so as to optimize the combined treatment plan [139].

It is noteworthy that a standardized PDO efficacy evaluation system is urgently needed for the clinical transformation of ferroptosis-targeted therapy. Existing studies showed that the PDO model of gastric cancer can accurately reflect the metabolic response of primary tumors to GPX4 inhibitors through histochemical staining and drug sensitivity test, which provides a technical basis for the development of dynamic monitoring programs based on lipid peroxidation markers (such as MDA and 4-HNE) [140]. In addition, the integration of nano-drug delivery systems with PDO platforms has become a new direction to break through the limitations of bioavailability. For example, iron-doped metal-organic framework nanoparticles can simultaneously enhance the tumor targeting of ferroptosis inducers and the permeability efficiency in the PDO model, providing an innovative strategy to solve the off-target effects of drugs [141].

We believe that the PDO platform can be crossed over in the future at the following levels. First, a cross-cancer and multi-omics integrated PDO biobanking is established, and the ferroptosis susceptibility signature gene clusters are mined by machine learning. Secondly, a dynamic culture system was developed to simulate the adaptive resistance mechanism of cancer cells to ferroptosis during treatment. Third, promote the design of clinical trials under the guidance of PDO to verify the correlation between organoid drug susceptibility data and patient clinical outcomes. Only through the deep integration of systems biology and precision medicine can the targeted ferroptosis therapy be promoted from the laboratory to the clinic and the transformation vision of “individualized ferroptosis therapy” be finally realized.

## 5. Future Prospects

As the largest ecosystem on Earth, the ocean harbors unique biochemical diversity and provides unprecedented molecular resources for ferroptosis-targeted therapy. In recent years, marine natural products have shown unique molecular mechanisms and therapeutic potentials in regulating the key pathways of ferroptosis, especially the lipid peroxidation and GPX4 signaling axis. For example, sponge-derived polyketones inhibit phospholipid synthesis [34] through mitochondria-specific lipid peroxidation [34,43]. These findings not only reveal the chemical specificity of marine compounds in the regulation of ferrodeath but also lay the theoretical foundation for their application in cancer therapy. Notably, some marine polyphenols, such as alginic acid derivatives derived from brown algae, can not only act as ferroptosis inducers to selectively kill tumor cells but also protect normal tissues by scavenging lipid free radicals. This dual function provides new ideas for overcoming the toxic side effects of traditional chemotherapy drugs [142].

From the perspective of mechanism, the ability of marine compounds to regulate the TME is particularly remarkable. For example, polyketides derived from deep-sea sponges can reduce the immunosuppressive function of TAMs and enhance anti-tumor immune responses by inhibiting their lipid metabolic reprogramming [143]. In addition, some marine-derived ω-3 polyunsaturated fatty acids, such as eicosapentaenoic acid EPA, can remodel the lipid metabolism phenotype of tumor cells, making them significantly more sensitive to ferroptosis inducers [43,116]. These findings suggest that marine natural products not only directly act on the core ferroptosis pathway but also may achieve synergistic anti-tumor effects by regulating the metabolic–immune interaction network in TME.

However, there are still several challenges in current research. First, the bioavailability of marine compounds needs to be addressed urgently. For example, although some polyphenols exhibit potent GPX4 inhibitory activity in vitro, they are susceptible to rapid metabolism or have difficulty penetrating tumor tissues in vivo [144]. To address this bottleneck, liposome-based or exosome-based nano-delivery systems may become an optimal strategy. For example, the encapsulation of sponge-derived polyketene compounds in pH-sensitive liposomes has significantly improved their accumulation efficiency at the tumor site [43]. Second, tumor-cell escape mechanisms from ferroptosis, such as activation of the FSP1-CoQ10 pathway, may attenuate the efficacy of marine compounds. Recent studies found that some red algae-derived terpenoids can inhibit both GPX4 and FSP1, thereby blocking the drug resistance pathway of tumor cells, which provides a direction for the development of multi-target ferroptosis inducers [105].

Future studies need to promote the development and application of marine ferroptosis regulators at multiple dimensions. Firstly, the integration of lipidomics and single-cell transcriptomics technology can accurately analyze the regulatory characteristics of marine compounds on the lipid metabolism network in tumor cells and their microenvironment. For example, lipidomic analysis revealed that some coral commensal metabolites could specifically reduce arachidonic acid (AA) content in tumor cell membrane phospholipids, thereby inhibiting ACSL4(1)-mediated lipid peroxidation. This finding provides a basis for the design of personalized treatment strategies targeting lipid metabolism [34]. Secondly, it is of great significance to explore the sequential combination strategy of marine compounds with conventional chemoradiotherapy. Preclinical studies showed that ascidium-derived alkaloids can significantly improve the killing effect of radiotherapy on drug-resistant tumors by enhancing radiation-induced mitochondrial lipid peroxidation, and this synergistic effect may be closely related to radiation-induced GPX4 degradation [55].

From the perspective of technological innovation, synthetic biology opens up a new path for the efficient preparation of marine ferroptosis regulators. For example, genetic engineering of the polyketo synthase gene cluster in marine actinomycetes enables the efficient biosynthesis of specific ferroptosis inducers, such as selenide compounds targeting GPX4 [145]. In addition, CRISPR-Cas9-based gene editing technology can be used to rapidly screen the targets of marine compounds. A recent study using genome-wide CRISPR screening technology found that green algae extracts indirectly affected GPX4 protein stability by regulating the MALT1 ubiquitination pathway, which not only revealed a new regulatory mechanism but also provided a molecular basis for optimizing the structure of marine compounds [105].

In terms of clinical translation, PDO models provide an ideal platform for the personalized screening of marine compounds. For example, using the PDO model of pancreatic cancer, it was found that some sponge-derived polyketo compounds could significantly enhance the sensitivity of tumor cells to ferroptosis by inhibiting the ACSL3-GPX4 protective axis secreted by pancreatic stellate cells (PSCs) [143]. This finding not only validates the therapeutic potential of marine compounds but also highlights the critical role of TME in the regulation of ferroptosis.

It is worth noting that there are still several scientific problems that need to be further explored in current research. For example, the molecular mechanisms underlying the bidirectional regulatory properties of marine compounds on ferroptosis (for example, some brown algae polyphenols can both induce ferroptosis in tumor cells and inhibit lipid peroxidation in normal cells) have not been fully elucidated. Recent studies showed that this selectivity may be due to the difference in the expression level of lipid metabolism enzymes (such as ACSL4(1)) and REDOX homeostasis between tumor cells and normal cells, but the specific regulatory network still needs to be further elucidated [34]. In addition, the dynamic effects of marine ferroptosis regulators on the immune microenvironment (such as the temporal and spatial relationship between ferroptosis-related DAMPs’ release and T cell infiltration) still need to be systematically studied.

Based on the current research progress, we believe that future exploration should be strengthened in the following directions. The first is to develop a “ferroptosis metabolic profile” based on multi-omics data to predict the efficacy of marine compounds in different tumor subtypes. For example, tumors with high ACSL4(1) expression and low GPX4 activity may be more sensitive [43,105]. Second, the combined administration regimen of marine compounds and Programmed Cell Death Protein 1 (PD-1)/PD-L1 inhibitors is designed to take into consideration the synergistic effect of ferroptosis and immune checkpoint inhibitors. Preclinical evidence showed that some marine polysaccharides can indirectly inhibit GPX4 expression and promote lipid peroxidation in tumor cells by enhancing CD8+ T cell-mediated IFN-γ secretion, which provides theoretical support for immune-ferroptosis combined therapy [49].

Finally, we believe that the development of marine ferroptosis modulators should focus on “spatio-temporal specificity”. For example, prodrug-type marine compounds that are activated only at the tumor site can be designed by taking advantage of the low pH or high ROS conditions that are unique to the TME. A recent study combined sponge-derived selenide peptides with a pH-sensitive carrier to achieve tumor-specific activation [55]. In addition, to address the heterogeneity of metastatic tumors, the development of marine derivatives that can penetrate the blood–brain barrier or target tumor stem cells (such as some deep-sea fungi-derived iron-chelating agents) may also be a breakthrough direction [146].

## 6. Conclusions

Marine natural products, owing to their unique chemical diversity, offer new directions for cancer treatment by inducing ferroptosis in tumor cells through targeting key molecules like GPX4 and ACSL4(1), regulating lipid peroxidation and iron metabolism, activating immune responses, and reshaping the TME. However, their clinical translation faces challenges such as low bioavailability, tumor-type dependence, and tumor cell resistance mechanisms—including activating alternative lipid repair or up-regulating iron chelation pathways. Future research should leverage multi-omics technologies to deeply dissect their mechanisms of action, comprehensively elucidating their regulatory effects on lipid metabolic networks in tumor cells and their microenvironments. Additionally, developing highly selective and effective novel marine-derived compounds through structural modification and drug design to optimize pharmacokinetic properties for improved bioavailability and tumor targeting, combined with advanced delivery technologies and personalized screening platforms, is crucial to advancing their clinical application in cancer therapy.

## Data Availability

Not applicable.

## Abbreviations

The following abbreviations are used in this manuscript:

4-HNE	4-Hydroxynonenal
AA	Arachidonic acid
ACSL3	Long-chain acyl-CoA synthetase 3
ACSL4(1)	Long-chain acyl-CoA synthetase 4
AdA	Adrenal acid
ATGL	Adipose triglyceride lipase
ASMP(7)	Actin-derived small molecular peptide
BRD4	Bromodomain-containing protein 4
CFAs	Cyclic fatty acids
CoQ	Coenzyme Q
CRISPR-Cas9	Clustered Regularly Interspaced Short Palindromic Repeats-Cas9
CSCs)	Cancer stem cells
DAMPs	Damage-associated molecular patterns
DHA	Docosahexaenoic acid
DUBs	Deubiquitinizing enzymes
eIF	Eukaryotic translation initiation factor
EMT	Epithelial-mesenchymal transition
EPR	Enhanced permeability and retention
ETC	Electron transport chain
EPA	Eicosapentaenoic acid
FG-CDs@Cu	Functionalized carbon dots loaded with copper ions
FSP1	Ferroptosis suppressor protein 1
FTH1	Ferritin heavy chain
FXR	Farnesoid X receptor
GPX4	Glutathione peroxidase 4
GSH	glutathione
HDAC	Histone deacetylase
HMGB1	High mobility group box 1
iFSP1	Inhibitor of FSP1
KEAP1	Kelch-like ECH-associated protein 1
LIP	Labile iron pool
LPO	Lipid peroxides
MAPK	Mitogen-activated protein kinase
MALT1	Mucosa-associated lymphoid tissue lymphoma translocation protein 1
MDA	Malondialdehyde
mtROS	Mitochondrial reactive oxygen species
NPC	Nasopharyngeal carcinoma
Nrf2	Nuclear factor erythroid 2-related factor 2
NSCLC	Non-small cell lung cancer
PD-1	Programmed Cell Death Protein 1
PD-L1	Programmed Death-Ligand 1
PDO	Patient-derived organoids
PGE2	Prostaglandin E2
PPARγ	Peroxisome proliferator-activated receptor gamma
PSCs	Pancreatic stellate cells
PUFAs	Polyunsaturated fatty acids
RCD	Regulatory cell death
ROS	Reactive oxygen species
SAR	Structure-activity relationship
SCD1	Stearoyl-CoA desaturase 1
SDH	Succinate dehydrogenase
Sec	Selenocysteine
SPs	Sulfated polysaccharides
STING	Stimulator of interferon genes
TAMs	Tumor-associated macrophages
TfR1	Transferrin receptor 1
TGF-β	Transforming growth factor-β
TME	Tumor microenvironment
